# Editorial: Preparation, function and application of postbiotics

**DOI:** 10.3389/fmicb.2024.1491550

**Published:** 2024-09-30

**Authors:** Zhaojie Li, Chuantao Peng, Zhihong Sun, Ling Deng, Krych Lukasz

**Affiliations:** ^1^College of Food Science and Engineering, Qingdao Agriculture University, Qingdao, China; ^2^Special Food Research Institute, Qingdao Agricultural University, Qingdao, China; ^3^Key Laboratory of Dairy Biotechnology and Engineering, Ministry of Education, Inner Mongolia Agricultural University, Hohhot, China; ^4^Key Laboratory of Dairy Products Processing, Ministry of Agriculture and Rural Affairs, Inner Mongolia Agricultural University, Hohhot, China; ^5^Section of Food Microbiology and Fermentation, Department of Food Science, Faculty of Science, University of Copenhagen, Frederiksberg, Denmark

**Keywords:** postbiotics, metabolites, vesicles, sphingolipids, colorectal cancer, rheumatoid arthritis, ulcerative colitis, yogurt

The use of probiotics in healthcare areas is prevalent and has a long history. However, given the existed issues that viable probiotics face in terms of stability (intolerance to high temperature, oxygen, etc.), stress resistance (sensitivity to complicated food matrixes or *in vivo* gastrointestinal tract) (Li et al., 2019; Aponte et al., 2020), and safety (the use of probiotics in certain groups such as neonates and vulnerable populations, and the possible risk of transmitting antibiotic resistance genes) (Ohishi et al., 2010; Goldenberg et al., 2017; Imperial and Ibana, 2016), in recent years, more and more attentions have been payed to the inactive bacterial cells or their components, termed as postbiotics, making them a prospective alternative to active probiotics. And now, postbiotics have become a new frontier and hotspot in food and pharmaceutical research.

In 2021, the International Scientific Association of Probiotics and Prebiotics (ISAPP) defined postbiotic as “a preparation of inanimate microorganisms and/or their components that confers a health benefit on the host” (Salminen et al., 2021). Increasing evidence suggests that postbiotics are becoming valuable tools in combating human diseases. Animal and clinical trials have consistently demonstrated that postbiotics can positively impact immunity, support oral health, prevent osteoporosis, and fight against allergies, and so on (Dong et al., 2024). Certainly, the functions of postbiotics extend well beyond these. Xie et al. reviewed the resisting colorectal cancer (CRC) activity of postbiotics and the mechanisms of its action. It is suggested that intestinal microbial species are strongly associated with the development of CRC, showing increased abundance of pro-inflammatory opportunistic bacteria and decreased abundance of beneficial bacteria. This disorder would destroy the intestinal mucosal barrier, regulate the cell cycle of CRC tumor cells, promote CRC proliferation and metabolism, reprogram the tumor immune microenvironment, cause DNA damage, trigger inflammatory responses, induce gene mutations and alter the resistance to tumor chemotherapy, and so on. The mechanisms of anti-CRC of postbiotics are multifaceted, including regulating intestinal microbiota, enhancing intestinal mucosal barrier function, regulating immune response and regulating systemic metabolism. Ying et al. provided an overview of the self-limiting autoimmune disease, rheumatoid arthritis (RA), summarizing its etiological factors, and reviewing the beneficial effects of postbiotics on RA. One significant cause of RA is intestinal dysbiosis, an imbalance that can lead to inflammation and ultimately speed up the progression of the disease. The involvement of intestinal microbiota in the development of RA is primarily reflected in mucosal immunity and is linked to the differentiation of T cells, particularly regulatory T cells (Treg) and helper T (Th) cells. Therefore, the intestinal microbiota is an important target for treating rheumatoid arthritis. Postbiotics have been considered as a novel supplement for managing RA. Administering postbiotics orally can boost immunity, regulate the intestinal microbiota, and strengthen the intestinal mucosal barrier function. Moreover, metabolites produced by the intestinal microbiota can enhance the integrity of the intestinal barrier and regulate the Treg/Th17 cell balance, leading to reduced serum IL-17 levels and accelerated bone repair. Diabetic retinopathy (DR), a prevalent microvascular complication in diabetic patients, is commonly associated with gut dysbiosis. As an emerging strategy for treating DR, the beneficial effects of postbiotics on DR were reviewd by Chen et al. Numerous animal studies have shown that postbiotic intervention lowers hyperglycemia, reduces damage to retinal peripapillary and endothelial cells, improves retinal microcirculatory dysfunction, and thereby slows the progression of DR. Postbiotics function through systemic reactions *in vivo* and local reactions in the intestinal lumen, such as balancing the intestinal microbiota, boosting human immunity, and regulating physiological functions by crossing the intestinal barrier.

Like probiotics, the health benefits of postbiotics are also strain-specific. Therefore, animal and clinical trials have been used to screen for new postbiotic products. Moreover, it is important to compare the health benefits of active probiotics with those of their corresponding postbiotics. Bu et al. investigated the effects of *Lacticaseibacillus rhamnosus* 2016SWU.05.0601 (Lr-0601) and its postbiotics on male Kunming mice with dextran sulfate sodium salt (DSS)-induced ulcerative colitis (UC). The results showed that supplementation with both Lr-0601 and its postbiotics can effectively alleviate DSS-induced UC in mice by reducing colonic mucosal damage, down-regulating the levels of pro-inflammatory cytokines, up-regulating tight junction proteins. More importantly, postbiotics offer several advantages, including greater stability and enhanced safety compared to viable probiotics. The results of this study support that postbiotics can be a promising alternative to probiotics to be applied in the prevention and treatment of UC. A prospective, double-blind, placebo-controlled, randomized, parallel study was conducted on females by Motei et al.. The study assessed the impact of dietary supplementation with a postbiotic extract derived from *Bifidobacterium breve* BB091109 on levels of pro-inflammatory cytokines (CRP, IL-6, IL-10, TNF-α, and IFN-γ) and markers of endocrine function (DHEA, estradiol, estriol, progesterone, cortisol, and human growth hormone). The findings indicated that supplementation with this *B. breve*-derived postbiotic could improve endocrine function in women over 40 and promote beneficial changes in inflammatory markers.

As we know, postbiotics possess various functional activities, which might be due to their source bacterial strains, different composition and preparation methods. The active components responsible for the functions of postbiotics include inactivated bacteria, and bacterial fractions (cytosolic polypeptides, phosphoglycolic acids, peptidoglycans, teichoic acids, and surface proteins), etc. What needs to be highlighted is that metabolites can certainly be part of a postbiotic preparation, but they are not essential components of a postbiotic product (Vinderola et al., 2024). Recently, researchers have found some new active components in postbiotics. Bleibel et al. reviewed the role of bacterial extracellular vesicles in exerting the benefits of psychobiotics for neuropsychiatric treatment. It has been found that extracellular vesicles from psychobiotics can be absorbed from the gastrointestinal tract, reach the brain, and deliver intracellular contents to exert beneficial effects in multiple directions. These vesicles seem to enhance the expression of neurotrophic molecules, improve serotonergic neurotransmission, and possibly provide astrocytes with glycolytic enzymes. Sphingolipids, present in both higher animals and prokaryotes, are a class of lipids distinguished by their long-chain bases, which serve as the backbone, and feature an amine group along with two or three hydroxy groups at one end of their structure. The impact of microbial sphingolipids on host health was reviewed by Bai et al. Intestinal microbial sphingolipids can migrate from the gut to various host organs, positively affecting the immune system and metabolism. The administration of these sphingolipids to mice has been shown to produce an anti-inflammatory effect and decrease the number of colonic NKT cells. It has been shown that sphingolipids within outer membrane vesicles (OMVs) of *Bacteroidetes* act as agonists for TLR2 signaling in macrophages, thereby playing a key role in mitigating inflammatory signaling. On the other hand, the preparation process of postbiotics, and fermentation conditions determine the composition of their active ingredients, which in turn define their specific functionality. Nealon et al. investigated and compared metabolite profiles of postbiotics prepared with three lactic acid bacteria strains (*Limosilactobacillus fermentum, Lacticaseibacillus paracasei, Lacticaseibacillus rhamnosus*) cultured with and without rice bran. It was found that postbiotics prepared from rice bran fermented by *L. fermentum* and *L. paracasei* exihibited stronger antibacterial activity against *S. Typhimurium* than their respective probiotic-alone postbiotics. Non-targeted metabolomics analysis revealed that many antibacterial metabolites in *L. fermentum* and *L. paracasei* rice bran postbiotics increased significantly. Sadighbathi et al. investigated the impact of supplementing postbiotics derived from *Streptococcus thermophilus* (ST) and *Lactobacillus delbrueckii* subsp. *bulgaricus* (LB) in cheese whey (CW) and skim milk (SM) on the antioxidant activity, viability of yogurt starter cultures, and quality parameters of low-fat yogurt during storage. The results showed that The LB-CW (*Lactobacillus bulgaricus* postbiotic-containing cheese whey) sample exhibited the highest antioxidant activity, and the LB-CW and LB-SM yogurt samples exhibited significantly higher body and texture scores. Tong et al. developed a solid-state fermentation preparation method for postbiotics with increased antimicrobial, antioxidant, and anti-inflammatory activities.

There is a broad consensus that the metabolic changes in microorganisms are regulated by genes. By analyzing the whole genome of the source strain and optimizing fermentation conditions to regulate the expression of certain functional genes, it may be a good strategy for preparing a postbiotic with specific function. Wu et al. analyzed the whole genome of *Bacillus subtilis* BS21 derived from pig feces and identified seven gene clusters involved in antimicrobial biosynthesis of secondary metabolites. They utilized response surface methodology (RSM) to optimize the medium components and fermentation parameters for antimicrobial secondary metabolite production by strain BS21. As a result, the production of antimicrobial secondary metabolites by BS21 was increased by 43.4%. Fang et al. investigated the changes and effects of fermented milk metabolites in mutant strains of *L. paracasei* following the knockout of the *ldh* gene. The results indicated that all differential metabolites in the mutant strain were upregulated (*P* < 0.05), including amino acids and their precursors, acetyl coenzyme A, and other metabolites involved in amino acid and fatty acid synthesis, all of which are linked to the formation of fermented milk flavor. The data established a connection between the *ldh* genes and strain growth and metabolism, offering potential targets for regulating flavor compounds in fermented milk.

With this Research Topic, we have gathered a collection of 12 research articles that offer fresh perspectives on various aspects of postbiotic research. These studies explored the multifunctional roles of postbiotics and underscored their promising potential for applications in healthcare.
